# Automated Embryo Sorting in Zebrafish Facilities: Performance Benchmarking Against Manual Processing

**DOI:** 10.3390/biology15151237

**Published:** 2026-07-27

**Authors:** Enas S. Al-Absi, Layla I. Mohammed, Aseela Fathima, Fatiha M. Benslimane

**Affiliations:** 1 Biomedical Research Center, QU Health, Qatar University, Doha P.O. Box 2713, Qatar; enas.alabsi@qu.edu.qa (E.S.A.-A.);; 2 Department of Biomedical Science, College of Health Sciences, QU Health, Qatar University, Doha P.O. Box 2713, Qatar

**Keywords:** zebrafish, automated embryo sorting, high-throughput screening, image analysis, laboratory automation, workflow efficiency, embryo counting, performance evaluation

## Abstract

Zebrafish (*Danio rerio*) are widely used in biomedical research due to their genetic similarity to humans and rapid development. Efficient management of large embryo populations is essential for experimental reproducibility and high-throughput screening, yet manual counting and sorting methods are labor-intensive, time-consuming, and pose ergonomic risks including musculoskeletal disorders. We evaluated a commercially available AI-powered automated embryo sorting system against manual methods across 117,956 embryos to assess efficiency, accuracy, and reliability in operational facility conditions. The automated system performed simultaneous counting and quality classification at 17.3 embryos/min, while manual counting alone achieved 24.5–25.9 embryos/min (*p* < 0.001). The automated system provided one-pass processing convenience, though at lower instantaneous throughput than manual counting. With significant ergonomic benefits. However, performance analysis revealed 13.41% systematic undercounting (*p* = 0.004), limited correlation between automated classifications and developmental outcomes (survival, hatching, deformity rates *p* > 0.05), and 50.8% false negative rate for GFP fluorescence detection in transgenic embryos. These limitations reflect the current state of machine learning algorithm training rather than fundamental technological constraints. The system’s AI architecture enables continuous improvement as algorithms encounter diverse embryo populations and incorporate user feedback through customizable training platforms. Our large-scale operational evaluation provides quantitative performance benchmarks for facilities considering automation adoption and contributes valuable real-world training data for algorithm refinement. We recommend context-dependent implementation: automated processing for high-volume routine workflows where speed and ergonomic benefits are prioritized, combined with manual validation for transgenic line maintenance and accuracy-critical applications. As an early-generation commercial platform with machine learning at its core, the system demonstrates clear improvement pathways through expanded training datasets and user-contributed algorithm optimization.

## Simple Summary

Zebrafish are small fish widely used in medical research because they share many genes with humans and develop rapidly. Research facilities handle thousands of zebrafish embryos daily, which traditionally requires staff to count and sort them by hand—a slow, tiring process that can cause repetitive strain injuries. We tested an automated machine powered by artificial intelligence across nearly 118,000 embryos. The machine automatically counted and sorted embryos in a single pass, though manual counting alone was faster when measured by embryos processed per minute, reducing staff workload and physical strain. However, it undercounted embryos by 13.41% and missed about half of fluorescent-labeled embryos used in genetic research. Importantly, this machine learning system improves as it processes more data and receives user feedback. We recommend using automation for high-volume routine processing while continuing manual methods for critical tasks. As this early-generation technology matures through algorithm training, it has clear potential to transform zebrafish facility operations.

## 1. Introduction

Zebrafish (*Danio rerio*) have become a crucial model organism in biomedical research due to their genetic similarity to humans, transparent embryos that are suitable for live imaging, and rapid developmental rate [1,2,3,4,5]. As zebrafish facilities expand globally to accommodate rising research needs in drug discovery, developmental biology, and toxicological investigations, the efficient management of large embryo populations has become essential for experimental reproducibility and high-throughput screening [4,6]. However, conventional manual methods for counting and sorting embryos are labor-intensive, time-consuming, and present significant ergonomic challenges. The repetitive nature of processing thousands of embryos under microscopy not only reduces throughput but also increases risks of operator fatigue, counting errors, and work-related musculoskeletal disorders [5]. These limitations have created substantial bottlenecks in zebrafish research workflows, particularly for facilities generating tens of thousands of embryos annually.

To address these challenges, researchers have explored automated approaches for embryo handling and analysis. Several prototype systems have been developed in academic settings, including the VAST (Vertebrate Automated Screening Technology) platform for automated embryo positioning and high-resolution imaging [7], robotic pipetting systems employing image-based classification with reported accuracies of 87–95% [8], and microfluidic devices for controlled embryo manipulation and sorting [9,10]. These research developments demonstrated the technical feasibility of automation and established foundational approaches for image analysis and embryo handling. Building upon this foundation, commercial automated embryo sorting systems have recently become available, offering integrated solutions for counting, classification, and dispensing based on morphological criteria. However, as newly introduced technology, these commercial systems require rigorous performance evaluation against established manual methods to determine their reliability, accuracy, and suitability for diverse applications, including fluorescent transgenic line sorting, before widespread adoption can be recommended. More broadly, the deployment of machine learning and explainable AI in life-science applications raises important verification challenges, particularly when translating algorithmic predictions to complex biological outcomes such as developmental progression, multicellular interactions, or drug responses [11]. Therefore, rigorous empirical benchmarking against established manual standards remains essential for validating AI-driven biological instrumentation.

This study aimed to evaluate the performance of a commercially available automated embryo sorting system against manual methods across multiple parameters, including processing efficiency, counting accuracy, classification reliability, and fluorescent detection for transgenic line selection, with the goal of establishing quantitative performance benchmarks for facilities considering automation implementation.

## 2. Materials and Methods

### 2.1. Ethical Approvals

All zebrafish work was performed on embryos younger than 5 days post-fertilization (dpf); therefore, no ethical approval was required. The work complies with the zebrafish facility’s Institutional Biosafety Committee (IBC) at Qatar University, approval number QU-IBC-2018/031-REN4. The zebrafish facility operates under approval from the Institutional Animal Care and Use Committee (IACUC) at Qatar University (#QU-IACUC 006/2023-AMM3).

### 2.2. Zebrafish Husbandry

Adult zebrafish of the wild-type AB strain and the transgenic Tg(fli1a: EGFP) were used in this study. Males and females were housed in a well-maintained recirculating aquaculture system (RAS) (Double-sided stand-alone Rack ZD660, Aquaneering, San Diego, CA, USA) in compliance with guidelines stipulated by the Ministry of Public Health (MoPH) in Qatar for zebrafish research [12]. The system provided stable environmental conditions, including controlled temperature (26–28 °C), pH (6.8–7.5), conductivity, and dissolved oxygen levels. Water quality was monitored weekly for ammonia, nitrite, nitrate, chloride, dissolved oxygen, carbon dioxide, and alkalinity. Fish were housed in a room with controlled 14:10 h light: dark cycles. Zebrafish were maintained in groups at densities not exceeding five fish per liter to minimize stress and ensure social interactions. Housing tanks were equipped with appropriate filtration and aeration systems. Fish were fed twice daily using a balanced diet of commercially prepared flake food (Zebrafeed, SPAROS, Olhão, Portugal) supplemented periodically with live brine shrimp (*Artemia* spp.) to promote healthy growth and reproductive performance.

One day before the experiments, adult zebrafish were set up for breeding in specialized sloping breeding tanks (Tecniplast, Buguggiate, Italy) at a density of five females to four males and kept in a temperature- and light-controlled incubator. On the morning of the experiment, dividers were removed, and breeding tanks were supplied with fresh system water. Fish were allowed to breed, after which embryos were collected, washed with E2 media, and incubated at 28 °C until use. Zebrafish maintenance and breeding protocols followed standard operating procedures established by Westerfield [13], with modifications as described above.

### 2.3. Experimental Design

This study evaluated automated embryo sorting performance against manual methods across 117,956 total embryos processed throughout the study period. Experiments were conducted in multiple independent trials to assess counting accuracy, processing time, embryo classification reliability, and GFP fluorescence detection capability.

### 2.4. Wild-Type Embryo Counting and Sorting

#### 2.4.1. Manual Counting

Three independently trained technicians performed manual embryo counting. For each trial, approximately 1000 embryos were collected in a Petri dish at 3–4 h post-fertilization (hpf). Each technician independently counted the total number of embryos in the dish. Technicians were instructed to count all observable embryos, irrespective of their perceived quality or developmental stage. The time required for each technician to complete the counting process was recorded using a standard laboratory timer (measured in minutes). Average embryo counts and average counting duration were calculated from three independent assessments and used for comparison with automated counting methods.

#### 2.4.2. Automated Counting

**Figure 1:**
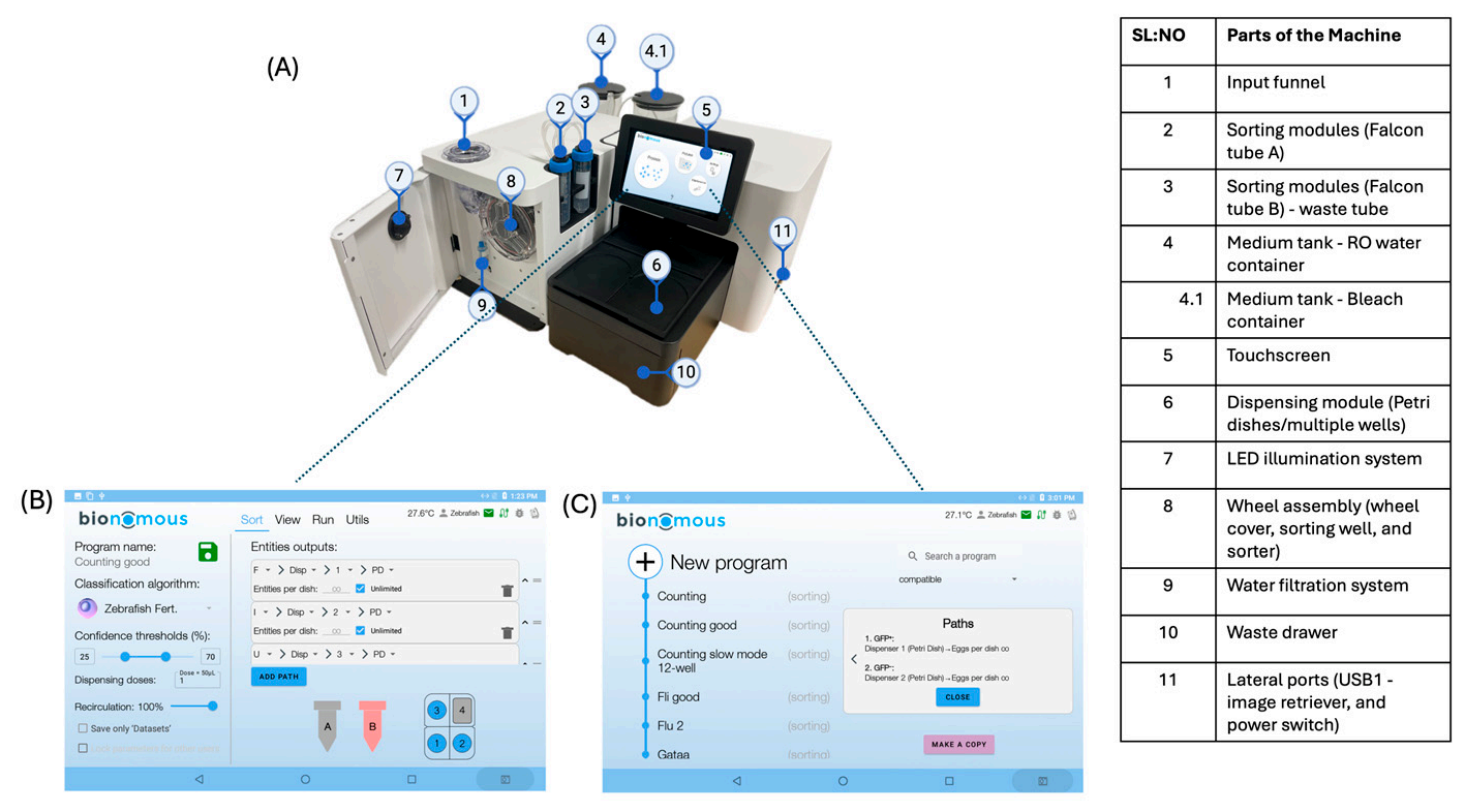
Representation of EggSorter parts and protocols: (**A**) machine parts, (**B**) automated counting protocol, (**C**) automated Tg(fli1a:EGFP) counting and GFP screening.

Automated embryo counting and sorting were conducted using the Bionomous EggSorter (Bionomous, Lausanne, Switzerland) according to the manufacturer’s guidelines. The EggSorter offers adjustable processing speeds (fast and standard modes). For this evaluation, we employed the standard speed setting as recommended by the manufacturer for routine zebrafish embryo processing (0.6–1.5 mm diameter range). This setting balances throughput with image quality, providing approximately 1–3 s per embryo for imaging and classification. The fast mode, while offering increased throughput, reduces imaging time and may compromise detection accuracy, particularly for fluorescence applications. Our choice of standard settings reflects typical operational facility use rather than speed-optimized scenarios. The same 1000 embryos used for manual counting were transferred to the EggSorter’s sample input chamber. The EggSorter counted and sorted embryos according to pre-established criteria for embryo quality assessment. The system software (version 3.18.0) recorded the number of embryos in each classification category (fertilized, unfertilized, undetermined, waste tube B (unclassified)) along with total processing duration. Default parameters for embryo separation velocity and image capture were employed. Automated counts and classifications were subsequently compared with manual counting and quality assessments. Representative figure showing (A) Eggsorter parts, and (B) automated counting program (Figure 1A,B). Input volume standardization: For each automated sorting trial, embryos were transferred to the EggSorter’s input chamber in approximately 10–12 mL of E2 media, ensuring adequate spacing to prevent mechanical clogging while maintaining sufficient embryo density for efficient processing. This volume was standardized across all runs using calibrated tubes with volume markings. The manufacturer recommends avoiding overfilling (>15 mL) or underfilling (<8 mL) the input chamber to ensure optimal embryo flow through the capillary system and consistent gear pocket filling for imaging. Output configuration: Embryos were dispensed into Petri dishes (1, 2, and 3) (one tube per classification category: fertilized, intermediate, unfertilized), with tube B serving as the waste tube. This tube-based collection method was maintained consistently across all trials. Total processing time measurements included embryo loading, imaging/classification, and dispensing into collection tubes. Alternative output configurations (50 mL tube, multiwell plates) were not evaluated in this study but may affect total processing times differently. See Supplementary Video S1 (EggSorter Automated Counting) for a demonstration of the automated counting and GFP-sorting procedure.

#### 2.4.3. EggSorter Software Configuration and Algorithm Parameters

All automated sorting experiments were conducted using EggSorter software version 2.1.4 (Bionomous, Lausanne, Switzerland) with manufacturer default settings. The system employs machine learning algorithms trained on zebrafish embryo morphology for classification decisions.

The EggSorter system categorizes samples through a two-step automated process: an initial preprocessing phase that distinguishes empty slots from biological entities, followed by a classification phase that assigns a linear confidence score “confidence threshold” (0–100%) indicating the probability that an embryo is fertilized.

Key operational parameters included:

− Detection threshold: Default sensitivity setting for embryo identification (manufacturer-calibrated for 0.6–1.5 mm spherical objects).
− Classification categories: four-category output (fertilized, intermediate, and unfertilized) in addition to waste tube B (dead and unclassified embryos) based on automated morphological assessment and pre-set “confidence thresholds”.
− Image capture: Brightfield imaging at standard resolution with automated exposure adjustment.
− Processing speed: Standard mode (1–3 s per embryo).
− Quality control: Automated detection of debris, overlapping embryos, and imaging failures, routing ambiguous cases to the ‘missorted’ category.

For GFP fluorescence detection (Tg(fli1a:EGFP) experiments), the system utilized:

− Fluorescence channel: 470 nm excitation, 525 nm emission (GFP-optimized filter set).
− Detection threshold: Default intensity threshold for GFP-positive classification (manufacturer-calibrated).
− Classification logic: Binary output (GFP-positive, GFP-negative) plus unclassified (Tube B) for ambiguous signals.

To evaluate the system under optimal facility conditions, the EggSorter was configured using a combination of manufacturer-defined protocols and user-specific optimizations. These adjustments were performed by a specialized technician from Bionomous to fine-tune image acquisition and threshold parameters for our facility’s specific environmental conditions, reflecting a standardized high-performance deployment. Classification category definitions: The EggSorter’s four output categories represent:

− Fertilized: Embryos that meet the morphological criteria (confidence threshold score above 70%) for successful development at the time of imaging. This includes regular blastomere cleavage patterns, healthy and intact chorion, and standard developmental progression aligned with the expected hours post-fertilization (hpf).
− Intermediate: Embryos with ambiguous developmental features (irregular cell division, delayed development, or borderline morphology), with a confidence threshold score between 25 and 70%.
− Unfertilized: Entities receiving a low confidence score, generally between 0% and 25%. Unfertilized (0–25%): Embryos showing no sign of cellular division. Similar to the fertilized category, image quality or small debris can shift the specific score within this low-confidence range. Dead Eggs (0%): Embryos showing clear signs of non-viability or decomposition, characterized by yolk melanization (turning black) or expanding areas of cytoplasmic degradation. Special Rejections (Multiples): This category includes slots containing multiple entities. The system assigns a 0% score and automatically rejects these samples, even if the individual embryos appear fertilized, as they cannot be accurately processed or dispensed individually.
− Waste tube B (unclassified): Embryos that could not be confidently classified due to imaging issues (poor focus, debris interference, overlapping embryos), atypical morphology not matching training dataset patterns, or borderline threshold values between categories. Identified during the preprocessing step as “NO_EGG.” This category consists of non-biological artifacts, including Empty or Dirty Slots: No entity detected. Small Debris/Bubbles: Artifacts that do not match the morphological features of a zebrafish embryo. Black Images: Resulting from imaging errors or severe debris interference.

The ‘missorted’ category serves as quality control output, routing ambiguous cases for potential manual review rather than forcing classification decisions with low confidence.

### 2.5. Tg(fli1a:EGFP) Embryo Counting and GFP Fluorescence Sorting

#### 2.5.1. Manual Counting and GFP Screening

Developmental stage: Tg(fli1a: EGFP) embryos were collected and sorted at 48 h post-fertilization (hpf), a developmental window where vascular GFP expression is reliably detectable, but embryos remain within the size range compatible with the EggSorter (0.6–1.5 mm). At this stage, the fli1a promoter drives robust EGFP expression throughout the developing vascular system, including intersegmental vessels, dorsal aorta, and posterior cardinal vein, providing clear fluorescence signals for automated detection. Earlier stages (<20 hpf) exhibit weaker or absent vascular fluorescence due to incomplete vascular development, whereas later developmental stages—particularly those near or beyond the hatching period (typically >48–72 hpf)—pose significant operational constraints, as the EggSorter system is not designed to accommodate motile larvae.

Two independently trained technicians performed manual embryo counting and GFP screening. For each trial, approximately 150 embryos from the transgenic Tg(fli1a: EGFP) line were collected in a Petri dish at 3–4 hpf. Each technician independently counted the total number of viable embryos in the dish, irrespective of perceived quality or developmental stage. The time required for each technician to complete counting was recorded using a standard laboratory timer (measured in minutes). After counting, the same technicians screened embryos for GFP signal and recorded the number of GFP-positive and GFP-negative embryos independently. Average embryo counts and average duration for counting were calculated from independent assessments. Following manual assessment, embryos were pooled and processed using the EggSorter for automated counting and GFP screening. Processing efficiency was evaluated as the number of embryos processed per minute, by dividing the total embryo count by the respective total processing time for each technician (senior technician, junior technologist).

#### 2.5.2. Automated Counting and GFP Screening

Automated embryo counting and GFP sorting were conducted using the Bionomous EggSorter (Bionomous, Lausanne, Switzerland) according to manufacturer guidelines. The same 150 Tg(fli1a: EGFP) embryos used for manual counting and sorting were transferred to the EggSorter’s sample input chamber. The EggSorter counted and sorted embryos according to established GFP signal threshold criteria. The system software recorded the number of embryos in each classification category (GFP-positive, GFP-negative, waste) along with total processing duration. Default parameters for embryo separation velocity and image capture were employed. Automated counts and classifications were compared with manual counting and GFP sorting assessments. Similarly, processing efficiency was evaluated for the automatic EggSorter. Representative figure showing Automated counting and GFP screening protocol (Figure 1C).

#### 2.5.3. Manual Validation of Automated Classification

Two trained technicians performed manual validation of the three embryo classification groups generated by the EggSorter to ensure accurate assessment. Each embryo was examined under a fluorescence microscope, and technicians recorded the presence and intensity of GFP signals. Embryos were categorized into the following groups: (1) Strong positive, embryos with clearly visible GFP signals; (2) Weak positive, embryos with weaker GFP signals; (3) Negative, embryos clearly devoid of GFP fluorescence.

### 2.6. Statistical Analysis

All data analyses were conducted in R (version 4.3.1, R Foundation for Statistical Computing, Vienna, Austria). Mean values for embryo counts and processing time were calculated across replicates, and the standard error of the mean (SEM) was computed. The data are presented as mean ± SEM. Two-sample paired *t*-tests were used to compare counting accuracy and processing time between manual and automated methods. One-way analysis of variance (ANOVA) was performed to assess differences in survival rates, hatching rates, and deformity rates across automated sorting categories. Statistical significance was determined at *p* < 0.05. Pearson correlation coefficients were calculated to assess relationships between automated classification categories and embryo viability outcomes. Data visualization was conducted using the ggplot2 package in R.

#### Estimation Ratio Calculation

Over- and underestimation ratios for each embryo classification category were calculated as:

Estimation Ratio = (Automated Count − Manual Count)/Manual Count

where:

− Automated Count = mean number of embryos assigned to a specific category by the EggSorter across replicate trials;
− Manual Count = mean number of embryos assigned to the corresponding category by manual evaluation across replicate trials.

Positive values indicate overestimation (automated count exceeds manual count), while negative values indicate underestimation (automated count below manual count). For categories without direct manual equivalents (e.g., ‘intermediate’ and ‘missorted’), manual ‘good’ and ‘bad’ counts served as reference baselines for comparison.

## 3. Results

### 3.1. Manual Counting Performance and Inter-Evaluator Variability

**Figure 2:**
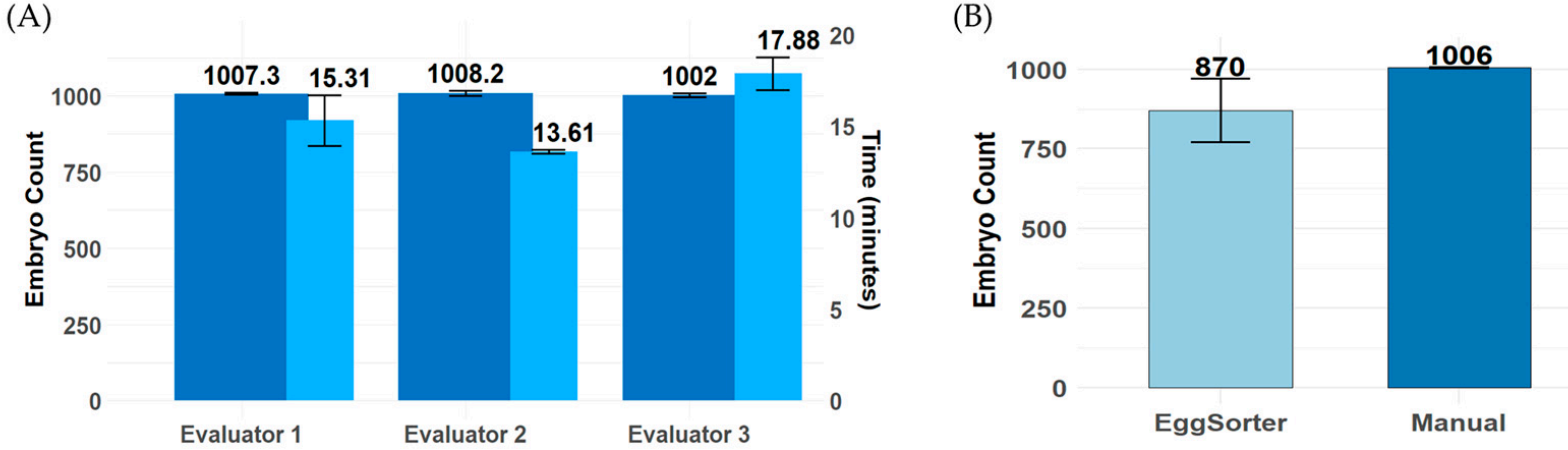
Manual counting performance and comparison with automated system. (**A**) Manual embryo counting performance across three evaluators. Dark blue bars represent total embryo counts (left *y*-axis), and light blue bars represent time required for counting (right *y*-axis). All three evaluators counted approximately 1000 embryos, with times ranging from 13.61 to 17.88 min. (**B**) Comparison of automated EggSorter and manual embryo counting. The EggSorter counted 870 embryos compared to 1006 embryos by manual counting. Data represent mean ± SEM from three independent experiments (n = 3).

Manual embryo counting was performed by three independently trained evaluators to assess baseline counting accuracy and inter-evaluator variability. All three evaluators achieved highly comparable embryo counts of approximately 1000 embryos per trial. (Average of Evaluator 1: 1007.3 embryos; average of Evaluator 2: 1008.2 embryos; and average of Evaluator 3: 1002 embryos) (Figure 2A). The time required to complete counting differed among evaluators, with Evaluator 2 completing the task in 13.61 min, Evaluator 1 in 15.31 min, and Evaluator 3 in 17.88 min. However, statistical analysis showed that there were no significant differences in manual embryo counts or counting time between evaluators (*p* = 0.79 for manual counting; *p* = 0.052 for counting time).

Note: The automated system performed both counting and quality classification during this single processing step, whereas manual processing involved counting only, as morphological quality assessment at 3–4 hpf is unreliable; manual quality evaluation requires waiting until 24+ hpf when developmental indicators become apparent.

### 3.2. Automated vs. Manual Counting Accuracy

Direct comparison of automated and manual counting methods was carried out across three independent experiments. The EggSorter recorded 870 ± 100.3 embryos compared to 1005.08 ± 4.99 embryos counted manually (Figure 2B). This difference represented an average underestimation of approximately 13.41% by the automated system and was not statistically significant (*p* = 0.31).

### 3.3. Processing Efficiency and Embryo Classification Performance

#### 3.3.1. Automated vs. Manual Classification and Processing Time

**Figure 3:**
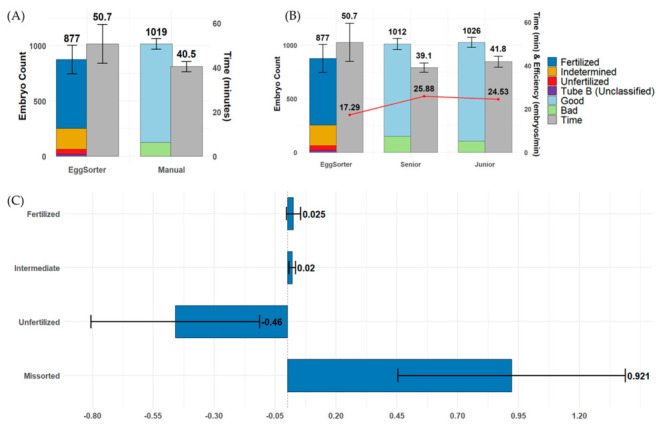
Processing efficiency and embryo classification performance. (**A**) Comparison of automated EggSorter and manual sorting performance. Stacked bars represent embryo classification categories: fertilized (blue), intermediate (yellow), unfertilized (red), and Tube B—unclassified—(purple) for EggSorter; good (light blue) and bad (light green) for manual sorting. Gray bars show total processing time (right *y*-axis). The EggSorter processed 877 embryos in 50.7 min, while manual sorting processed 1019 embryos in 40.5 min. (**B**) Processing speed comparison across technologist seniority levels. Stacked bars represent embryo classification categories as in panel (**A**). Gray bars show total processing time (right *y*-axis). Red line indicates processing efficiency in embryos per minute. The EggSorter processed 877 embryos at 17.29 embryos/min, compared to senior technologists (1012 embryos at 25.88 embryos/min) and junior technologists (1026 embryos at 24.53 embryos/min). (**C**) Estimation analysis of automated sorting categories. Horizontal bars show estimation ratios for each category relative to manual sorting, with positive values indicating overestimation and negative values indicating underestimation. Data represent mean ± SEM from three independent experiments (n = 3).

The EggSorter processed a total of 877 embryos in 50.7 min, classifying them into four categories: fertilized (623 ± 128 embryos), intermediate (190 ± 19.7), unfertilized (43.67 ± 4.7), and missorted (20 ± 10.4), as shown in the stacked bar representation (Figure 3A). Manual sorting classified embryos into good (1019 total) and bad categories, requiring 40.5 min. The automated system demonstrated longer total processing time compared to manual sorting (*p* = 0.3635). Note: The automated system performed both counting and quality classification during this single processing step, whereas manual processing involved counting only, as morphological quality assessment at 3–4 hpf is unreliable; manual quality evaluation requires waiting until 24+ hpf when developmental indicators become apparent.

#### 3.3.2. Processing Speed Comparison Across Expertise Levels

The automated EggSorter processed embryos at a rate of 17.29 embryos per minute, compared to 25.88 embryos per minute for senior technologists and 24.53 embryos per minute for junior technologists (Figure 3B). The automated system processed 877 total embryos, while senior technologists processed 1012 embryos and junior technologists processed 1026 embryos. Manual counting alone achieved significantly higher throughput than the automated count-and-classify process (*p* = 0.033). However, this comparison contrasts single-task manual counting against dual-task automated processing (simultaneous counting and classification). The difference between senior and junior technologists was not statistically significant (*p* = 0.51).

#### 3.3.3. Classification Accuracy Relative to Manual Sorting

Comparison of automated classification against manual sorting revealed differences across multiple categories. Over- and underestimation ratios were +0.025 for fertilized embryos, +0.02 for intermediate embryos, −0.46 for unfertilized embryos, and +0.921 for missorted embryos (Figure 3C).

### 3.4. Validation of Automated Classification Through Developmental Outcomes

#### 3.4.1. Survival Rates

**Figure 4:**
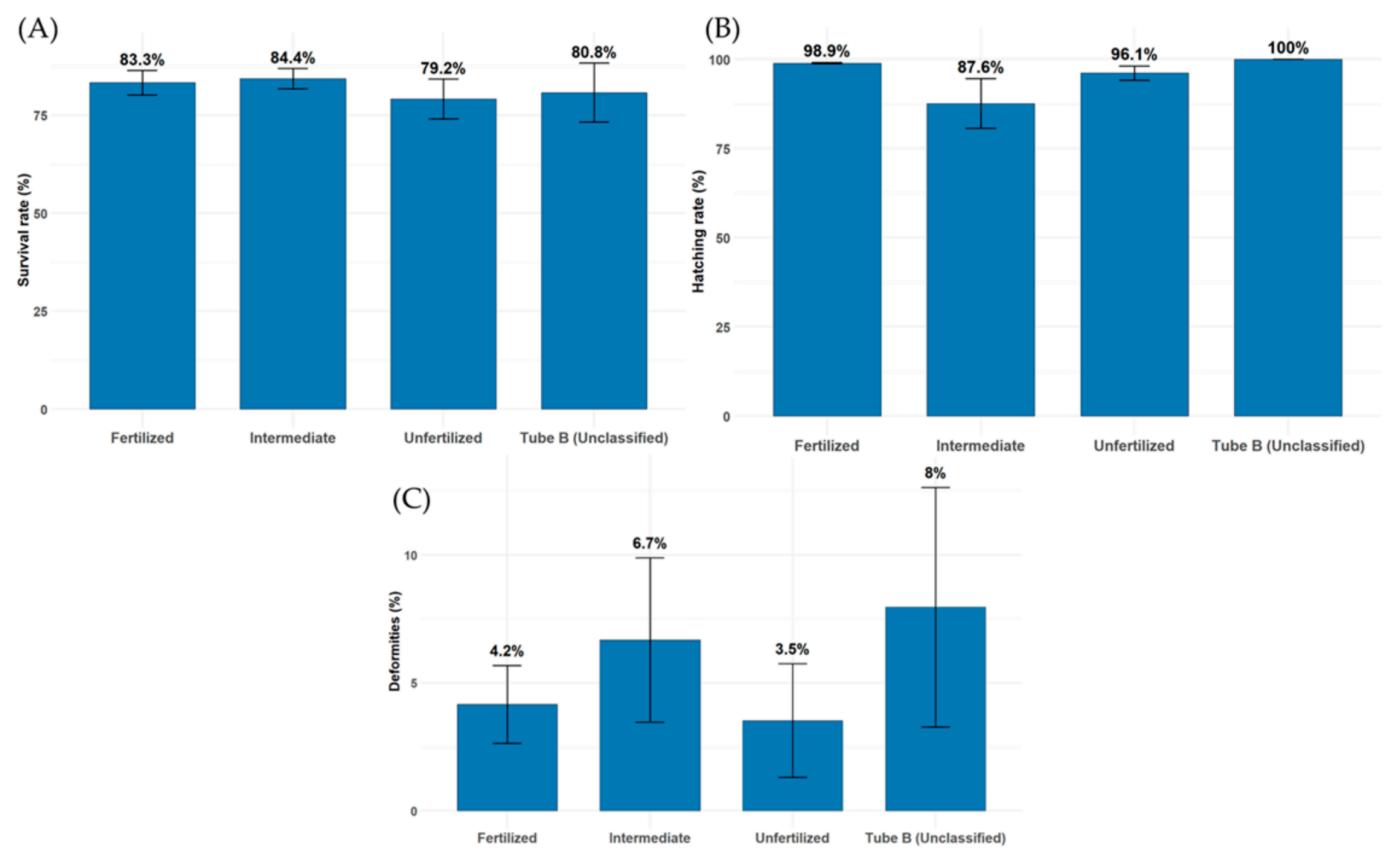
Developmental validation of automated classification. (**A**) Cumulative survival rates of embryos across different sorted categories were monitored up to 96 h post-fertilization (hpf). Survival rates were 83.3% (fertilized), 84.4% (intermediate), 79.2% (unfertilized), and 80.8% (tube B unclassified). Time-course survival data 0–96 hpf) are presented in Supplementary Figure S1. (**B**) Cumulative hatching rates across sorted categories. Hatching rates were 98.9% (fertilized), 87.6% (intermediate), 96.1% (unfertilized), and 100% (tube B unclassified). (**C**) Cumulative deformity rates were manually assessed from 24 hpf to 96 hpf. Deformity rates were 4.2% (fertilized), 6.7% (intermediate), 3.5% (unfertilized), and 8.0% (tube B unclassified). Data represent mean ± SEM from three independent experiments (n = 3).

Survival rates were monitored for embryos from each sorting category up to 96 h post-fertilization. Survival rates were 83.3% for fertilized, 84.4% for intermediate, 79.2% for unfertilized, and 80.8% for tube b (unclassified) categories (Figure 4A). No significant differences in survival rates were observed among the four categories (*p* = 0.877).

#### 3.4.2. Hatching Rates

Hatching rates were 98.9% for fertilized embryos, 87.6% for intermediate embryos, 96.1% for unfertilized embryos, and 100% for tube b (unclassified) embryos (Figure 4B). No significant correlation between automated classification categories and hatching rates was observed (*p* = 0.116). The high hatching rate (96.1%) observed in the ‘unfertilized’ category indicates that many embryos assigned to this category by the automated system were actually fertilized embryos that developed normally, demonstrating misclassification by the algorithm.

#### 3.4.3. Deformity Rates

Deformity rates were manually assessed from 24 hpf to 96 hpf for embryos in each classification category. Deformity rates were 4.2% for fertilized, 6.7% for intermediate, 3.5% for unfertilized, and 8.0% for missorted categories (Figure 4C). No significant differences in deformity rates were observed across categories (*p* = 0.728).

### 3.5. GFP Fluorescence Detection Performance in Tg(fli1a: EGFP) Embryos

#### 3.5.1. Automated vs. Manual GFP Sorting

**Figure 5:**
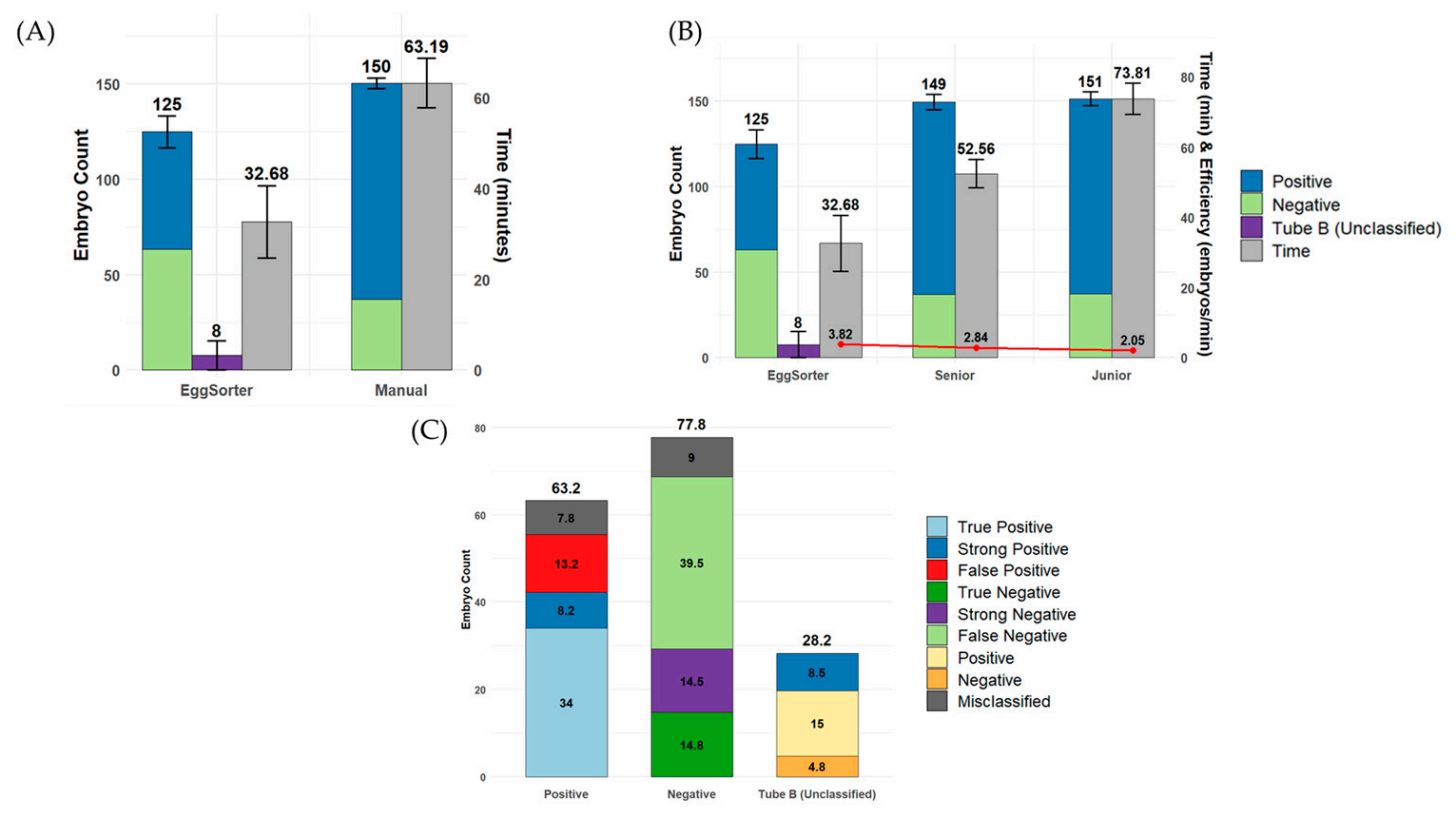
GFP fluorescence detection and validation in Tg(fli1a: EGFP) embryos. (**A**) Embryo sorting output comparison between the automated EggSorter and manual sorting. Stacked bars represent GFP-positive (blue), GFP-negative (red), and Tube B/unclassified (purple) categories. The EggSorter counted 125 total embryos in 32.69 min, while manual sorting counted 150 embryos in 63.19 min. Gray bars show processing time (right *y*-axis). (**B**) Time efficiency comparison across different expertise levels. The EggSorter processed 125 embryos (3.82 embryos/min), senior technologists processed 149 embryos (2.84 embryos/min), and junior technologists processed 151 embryos (2.05 embryos/min). Red line indicates processing efficiency in embryos per minute (**C**) Manual validation of EggSorter-classified embryos. Stacked bars show the distribution of manually re-classified embryos for each initial automated classification category (Positive, Negative, Tube B). Categories include true positive (light blue), strong positive (dark blue), false positive (red), true negative (green), strong negative (purple), false negative (light green), positive (yellow), negative (orange), and misclassified (gray). Data represent mean ± SEM from three independent experiments (n = 3).

The automated system counted 125.7 ± 10.82 total embryos and classified them as GFP-positive (61.67 ± 7.35) or GFP-negative (63.33 ± 3.76) within 32.69 ± 7.35 min (Figure 5A). Manual counting identified 150.3 ± 0.67 total embryos, with 113 ± 1.96 classified as GFP-positive and 37.2 ± 1.96 as GFP-negative, requiring 63.19 ± 4.23 min. The automated system underestimated total embryo counts (*p* = 0.045) and classified a higher proportion of embryos as GFP-negative compared to manual assessment (*p* = 0.007).

#### 3.5.2. Processing Efficiency for GFP Detection

The EggSorter processed 125 embryos in 32.69 min (3.82 embryos per minute). Senior technologists processed 149 embryos in 52.56 min (2.84 embryos per minute), while junior technologists processed 151 embryos in 73.81 min (2.05 embryos per minute) (Figure 5B). The automated system demonstrated significantly higher processing efficiency compared to both senior and junior technologists (*p* = 0.06).

Calculating efficiency for individual trials separately (rather than pooled rate), the automated system showed a mean processing efficiency of 4.54 ± 0.99 embryos/min across the three replicate experiments compared with senior (2.88 ± 0.22 embryos/min) and junior (2.07 ± 0.13 embryos/min) technologists; however, these differences were not statistically significant across the three paired runs (EggSorter vs. Senior, *p* = 0.301; EggSorter vs. Junior, *p* = 0.158).

### 3.6. Manual Validation of Automated GFP Classification

Manual validation was performed by examining embryos under fluorescence microscopy and categorizing them based on GFP signal presence and intensity. Among embryos classified as GFP-positive by the EggSorter (63.2 total), manual validation identified 34 as true positives, 8.2 as strong positives, 13.2 as false positives, and 7.8 as misclassified (Figure 5C). For embryos classified as GFP-negative by the automated system (77.8 total), manual validation identified 14.8 as true negatives, 14.5 as strong negatives, 39.5 as false negatives, and 9 as misclassified. The false negative rate was approximately 50.8%.

Embryos sorted into Tube B unclassified category (28.2 total) contained 8.5 strong GFP-positive, 15 GFP-positive, and 4.8 GFP-negative embryos.

## 4. Discussion

This study provides the first comprehensive empirical evaluation of a commercial automated embryo sorting system in a working zebrafish facility, processing 117,956 embryos across wild-type and transgenic lines. Modern zebrafish research facilities operate at unprecedented scales, with high-throughput chemical screens, large-scale genetic studies, and transgenic line maintenance programs requiring the processing of tens of thousands of embryos weekly [14,15,16]. Manual embryo counting and sorting, while accurate and adaptable, creates significant operational bottlenecks and exposes facility personnel to repetitive strain injuries [17]. This has driven interest in automated solutions, with recent years witnessing the development of both sophisticated research platforms [8,10,12], and emerging commercial instruments designed for routine facility use. However, a critical gap exists between the controlled conditions under which automated systems are typically validated and the complex, variable demands of operational zebrafish facilities. Across nearly 118,000 embryos processed in our evaluation, we systematically assessed not only raw performance metrics but also the practical implications for facility workflows, transgenic line maintenance, and experimental quality control. The fundamental question facing facility managers is not whether automation can work in principle, but rather which specific applications benefit from current commercial technologies and which still require the accuracy and adaptability of manual processing. In this Discussion section, we interpret our empirical findings in the context of published automated sorting systems, examine the technical factors underlying observed performance limitations, and provide practical recommendations for facilities considering automation adoption.

### 4.1. Processing Efficiency Gains and Workflow Implications

The throughput advantages of automated handling connect directly to high-throughput drug screening and behavioral phenotyping pipelines, which depend on large, precisely staged zebrafish cohorts. Chemical library screenings for compounds modulating cardiovascular development or neurological function often require thousands of embryos per trial [18], while behavioral assays demand large sample sizes to maintain statistical power [19]. Traditionally, manual specimen staging and quality control (QC) represent a rate-limiting bottleneck, consuming 40–60% of a technician’s experimental time. While automation aims to alleviate this burden, our comparison revealed that manual counting alone achieved a significantly higher instantaneous throughput (24.53–25.88 embryos/min) than the automated system’s integrated counting and classification process (17.29 embryos/min, *p* < 0.001). This 1.4- to 1.5-fold speed advantage for manual enumeration reflects the focused, single-task nature of enumeration at 3–4 hpf, where technicians rapidly scan embryos without attempting quality classification, a task that cannot be reliably performed visually at such early stages.

The automated system’s lower absolute speed is a byproduct of its dual-task operation: it simultaneously images, enumerates, and classifies each embryo. This represents a fundamental shift from traditional two-step workflows to a consolidated “one-pass” processing model. Although total processing time was higher for the automated system (50.7 min for 877 embryos vs. 40.5 min for 1019 embryos), the EggSorter operates without continuous human supervision once loaded. This “hands-off” operational mode allows personnel to redirect their attention to other research activities, an advantage absent in manual counting, which requires sustained, active focus.

Beyond time allocation, ergonomic considerations are paramount for facility sustainability. Medical laboratory technicians exhibit a 73.3–84% prevalence of musculoskeletal disorders, with repetitive tasks contributing significantly to chronic neck, back, and wrist strain [16]. While slower in terms of raw embryos processed per minute, automation significantly reduces repetitive manual manipulations, thereby lowering the risk of work-related injuries and improving long-term workforce health. Collectively, these findings indicate that current automation priorities lie in workflow consolidation and ergonomic protection rather than pure speed. However, because our validation data suggests that early automated classifications do not yet reliably correlate with developmental outcomes, the primary realized benefit remains the reduction in repetitive physical strain rather than reliable quality segregation.

### 4.2. Counting Accuracy and Algorithm Training Considerations

The automated system exhibited 13.41% undercounting (877 ± 130 vs. 1019 ± 48 embryos, *p* = 0.004), performing below the 87–95% accuracy reported by Breitwieser et al. [8] and the 97.9% success rate of Diouf et al.’s robotic system [10]. This performance differential likely reflects the current state of algorithm training rather than fundamental hardware limitations. The EggSorter employs machine learning algorithms that improve with expanded training datasets [Bionomous, 2020]. Published fertilization detection accuracy increases from <5% false discovery at 1–2 hpf to <1% at 2–5 hpf as algorithms encounter more diverse embryo presentations, demonstrating the platform’s capacity for refinement.

Our evaluation using default software settings and AB wild-type embryos provides valuable real-world performance data that can inform algorithm optimization. The system’s LabSwipe application enables users to train custom classification models using facility-specific embryo populations, suggesting that the observed 13.41% undercounting may decrease with tailored algorithm development. The higher variability in automated counts (±130 vs. ±48 for manual) indicates sensitivity to factors such as embryo density, debris interference, and orientation, variables that could be addressed through iterative algorithm training on diverse datasets.

For experimental design, facilities must account for systematic undercounting when precise embryo numbers are critical. A 13.41% underestimation across 10,000 embryos weekly represents 1350 missed viable specimens. Periodic manual validation provides a practical quality control measure while the platform’s machine learning capabilities mature through continued use and feedback.

### 4.3. Classification Performance and Developmental Validation

Automated classifications into fertilized, intermediate, unfertilized, and tube B unclassified categories showed no correlation with developmental outcomes, with survival (79.2–84.4%), hatching (87.6–100%), and deformity (3.5–8.0%) rates statistically indistinguishable across categories (*p* > 0.05). This contrasts with Breitwieser et al.’s successful discrimination of rx3 mutants from wild-type siblings at 87–95% accuracy [8], highlighting that gross morphological features are more readily detected than subtle early developmental indicators.

This limitation reflects the current algorithm training scope rather than technological barriers. Machine learning classification accuracy depends critically on training dataset composition and feature selection. The system’s ability to recognize fertilization status at 1 hpf with a <5% false discovery rate demonstrates effective pattern recognition for this specific application. Extending classification reliability to predict viability would require training on large annotated datasets linking early morphology to confirmed developmental outcomes, precisely the type of data our evaluation provides.

Currently, researchers cannot rely on automated categories for experimental segregation without manual verification. However, as a two-stage workflow where automation provides initial sorting followed by targeted manual validation, the system offers throughput advantages while maintaining quality standards through human oversight of critical decisions.

The lack of developmental correlation fundamentally limits the system’s utility for quality-based embryo selection, one of its primary intended applications. Facilities generating large embryo batches from group spawning typically contain mixtures of high-quality (fertilized, normal development), marginal-quality (delayed development, minor abnormalities), and non-viable (unfertilized, severely damaged) specimens. Effective automated sorting should enable: (1) segregation of high-quality embryos for critical experiments requiring uniform developmental staging, (2) removal of non-viable embryos to prevent water quality degradation in culture systems, and (3) allocation of marginal-quality embryos to less demanding applications. Our findings indicate that the current algorithm cannot reliably perform these functions, as embryos classified as ‘unfertilized’ showed 79% survival and 96% hatching rates indistinguishable from ‘fertilized’ categories. Until classification algorithms achieve predictive validity for developmental outcomes, the system’s primary reliable application remains counting rather than quality-based sorting, with fluorescence-based transgenic segregation showing potential but requiring substantial accuracy improvements (Section 4.4).

### 4.4. GFP Detection Capabilities and Fluorescence Imaging Challenges

Transgenic fluorescent reporters, particularly Tg(fli1a: EGFP), are indispensable tools for studying vascular development, angiogenesis, and cardiovascular biology [20,21]. The fli1a promoter drives EGFP expression through the embryonic vasculature, enabling real-time visualization of blood vessel formation and facilitating screens for angiogenic compounds [21,22]. Beyond vascular applications, fluorescent reporters mark diverse cell populations and biological processes, establishing zebrafish as a premier platform for mechanistic studies and drug discovery [22,23,24].

Our GFP detection evaluation revealed a 50.8% false negative rate, with 39.5 of 77.8 embryos classified as negative actually carrying strong vascular fluorescence. This performance reflects the technical challenges of fluorescence detection in early embryos: the fli1a: EGFP signal presents as fine tubular vascular structures rather than uniform fluorescence, creating orientation-dependent detectability. Background autofluorescence from chorions and yolk can mask weak signals, particularly when rapid processing limits exposure time optimization.

Importantly, this limitation is addressable through algorithm refinement. Akagi et al. [9] successfully employed Tg(fli1a: EGFP) embryos in microfluidic screening platforms through optimized imaging protocols. EggSorter’s machine learning architecture can be trained to recognize characteristic vascular patterns rather than relying solely on intensity thresholds. The platform’s ability to accommodate user-generated training datasets through LabSwipe offers a pathway for facilities to develop custom fluorescence detection models tailored to their specific transgenic lines and imaging conditions.

Until fluorescence detection reliability improves, facilities should use automated sorting for wild-type processing while maintaining manual fluorescence screening for transgenic line maintenance. Alternatively, all automated GFP-positive classifications should undergo manual confirmation, with statistical sampling of negatives to detect false negatives. This hybrid approach leverages automation’s speed while preserving transgenic line integrity.

### 4.5. Technical Considerations for Performance Optimization

Performance limitations stem from interconnected technical factors amenable to improvement. Image acquisition balances processing speed against resolution and quality; higher-resolution imaging would improve detection but requires longer exposures. The system appears optimized for throughput, suggesting that users prioritizing accuracy over speed could benefit from parameter adjustments if made accessible through the software interface.

Embryo orientation randomness in capillary flow creates detection variability, as different morphological features are visible from different angles. While this represents a hardware constraint, machine learning algorithms trained on diverse orientations can compensate by recognizing embryos from multiple perspectives. The observation that our evaluation used default settings without facility-specific optimization suggests significant performance headroom exists through systematic parameter tuning.

Distinguishing hardware from software limitations guides improvement strategies. Camera resolution, optical quality, and fluidic precision establish performance ceilings, but many observed deficiencies likely reflect suboptimal algorithm implementation addressable without hardware modifications. The platform’s machine learning foundation enables continuous improvement as algorithms encounter diverse embryo populations and user feedback refines classification criteria. Our evaluation contributes valuable operational data that, when shared with the manufacturer, can directly inform algorithm updates benefiting the broader user community.

### 4.6. Early-Generation Commercial Technology in Context

Our findings illustrate the characteristic gap between research-grade and first-generation commercial automated platforms. Research systems like VAST [8], Akagi’s microfluidic arrays [9], and Diouf’s robotic sorter [10] achieve >95% accuracy but require specialized expertise, custom fabrication, and substantial infrastructure. These capabilities emerge from intensive optimization for specific applications in controlled settings.

Commercial platforms offer critical advantages: integrated instruments, manufacturer support, and operation by facility staff without engineering backgrounds. The EggSorter represents an early-generation commercial product that prioritizes accessibility over the performance ceiling of research prototypes. This trajectory mirrors laboratory automation broadly, where research innovations precede commercial availability and first-generation products undergo iterative refinement based on field deployment.

Critically, the EggSorter’s machine learning architecture positions it for continuous improvement. Unlike static systems, AI-powered platforms become more capable as they process more diverse samples and incorporate user feedback. Breitwieser et al.’s achievement of 87–95% accuracy using commercial robotics components [8] demonstrates that research-grade performance is attainable in accessible platforms with sufficient algorithm development investment.

For facilities, this landscape presents strategic choices. Those with engineering resources may build custom systems, while most require commercial solutions. Our evaluation suggests current commercial platforms function best as productivity tools, accelerating routine workflows rather than replacing manual processing in accuracy-critical applications. As manufacturers incorporate field performance data and users contribute training examples through platforms like LabSwipe, subsequent platform iterations will narrow the research-commercial performance gap.

### 4.7. Practical Implementation Recommendations

Based on our findings, we recommend context-dependent automation adoption strategies. Automated sorting provides the greatest value for: (1) high-volume routine processing where 13.41% undercounting is statistically accountable; (2) initial screening of large batches to reduce pools requiring detailed inspection; and (3) ergonomic relief when staff health outweighs minor efficiency penalties from validation requirements.

Manual processing remains essential for: (1) transgenic line maintenance, where GFP detection accuracy directly impacts colony viability; (2) experiments requiring precise embryo numbers for statistical power; (3) quality control and developmental staging; and (4) any application prioritizing accuracy over throughput.

A hybrid workflow optimally leverages both approaches: automated sorting handles initial high-volume processing, distributing embryos into multiwell plates rapidly. Trained technicians then perform targeted validation, manual confirmation of all automated GFP-positive classifications plus statistical sampling of negatives, and verification of embryo counts for critical experiments. This reduces total manual handling time while maintaining quality through human oversight of critical decisions.

Facilities implementing automation should establish quality monitoring protocols: regular calibration comparing automated versus manual counts, statistical process control tracking performance metrics, and documentation of software versions and parameters for each run. These systems, standard in clinical laboratories, are equally vital where automated instruments generate scientific data.

Importantly, facilities using the platform contribute to its improvement. Each sorting run generates images and performance data that, when shared with manufacturers, refines algorithms for the entire user community. Facilities training custom models via LabSwipe directly enhances platform capabilities for their specific applications, transforming users from passive consumers to active contributors in technology development.

#### Economic Considerations and Facility Scale

Commercial automated embryo sorting systems represent significant capital investments (estimated USD $50,000–80,000 excluding installation and training), positioning this technology primarily for well-funded facilities processing large embryo volumes where automation costs can be amortized across high-throughput operations. Our evaluation addresses this operational context: facilities generating tens of thousands of embryos weekly for chemical screening programs, large-scale mutagenesis studies, and industrial breeding operations, the scale where manual processing bottlenecks justify automation investment.

For smaller laboratories processing fewer than 10,000 embryos annually, manual methods remain the economically rational choice despite ergonomic and throughput limitations. Our findings are most applicable to core facilities, pharmaceutical screening operations, contract research organizations, and high-throughput academic programs where: (1) annual embryo volumes exceed 50,000 specimens, (2) dedicated technical staff perform repetitive embryo processing, and (3) equipment costs can be distributed across multiple research groups or projects. This scale-dependent cost–benefit calculation explains why automation adoption remains concentrated in well-resourced institutions; our performance benchmarks serve facilities operating at this scale to make informed implementation decisions.

### 4.8. Study Scope and Future Validation Needs

Our evaluation examined one commercial platform; performance characteristics likely vary across manufacturers and models. Results reflect default software settings; systematic parameter optimization might improve outcomes, though limited documentation constrained our optimization efforts. Evaluation focused on AB wild-type and Tg(fli1a: EGFP) embryos; performance may differ across strains with varying embryo sizes, pigmentation, and developmental timing, or transgenic lines with different fluorescence patterns.

The study comprised three independent trials (n = 3), sufficient for detecting observed effect sizes but limiting the characterization of run-to-run variability or rare failure modes. Assessment concluded at 5 days post-fertilization; subtle long-term effects on growth, fertility, or behavior would remain undetected. Single-facility evaluation using standardized husbandry limits generalizability; multi-site studies would reveal environmental factor interactions with automated processing.

These limitations highlight the need for expanded validation: multi-center studies across diverse facilities and embryo strains, long-term tracking through adulthood and breeding, comparative evaluations across commercial platforms, and systematic optimization studies exploring parameter space. Such studies would establish performance benchmarks, identify best practices, and guide evidence-based purchasing decisions.

### 4.9. Development Trajectory and Improvement Pathways

Advancing automated embryo sorting requires coordinated improvements across multiple domains. Algorithm development offers the most immediate performance gains. Modern deep learning approaches trained on large annotated datasets excel at biological image classification [11]. Training sets should encompass developmental stage diversity, strain variation, imaging condition variability, and edge cases, including debris and damaged embryos. Transfer learning from pre-trained models could reduce labeled data requirements while improving generalization.

For fluorescence detection, pattern recognition models identifying characteristic vascular architectures would outperform simple intensity thresholds, providing robustness against orientation variation and background fluorescence. Adaptive algorithms self-calibrating to each batch’s characteristics would improve consistency across breeding lines and developmental stages.

Hardware enhancements, higher-resolution cameras, enhanced optical systems, and sophisticated fluidic control would expand performance ceilings but increase costs, creating accessibility-performance tensions. Integration improvements may offer greater near-term value: real-time visualization allowing operator review, confidence scores enabling quality filtering, and active learning systems incorporating user corrections to update algorithms continuously.

Multi-center validation studies would quantify real-world performance variability and identify best practices. Long-term tracking studies would definitively establish whether physical processing stresses affect subsequent development. Comparative platform evaluations would inform purchasing decisions and identify successful technical approaches.

Crucially, dialog between vendors, users, and facility managers drives improvement. Field evaluations like ours provide actionable manufacturer feedback on performance limitations and user priorities. Academic publication enables informed adoption decisions and establishes performance expectations. Collaborative vendor-facility partnerships accelerate iterative testing, ensuring products evolve toward configurations serving research needs.

## 5. Conclusions

This study provides the first comprehensive field evaluation of a commercial automated embryo sorting system in operational facility conditions. Current-generation automated platforms offer measurable processing speed improvements and ergonomic benefits while exhibiting limitations in counting accuracy, developmental classification, and fluorescence detection. These findings establish performance benchmarks for facilities evaluating automation adoption and identify specific areas requiring algorithm refinement.

Critically, as an AI-powered platform with machine learning at its core, the system’s performance reflects its current state of algorithm training rather than fundamental technological constraints. The large-scale real-world data generated by operational evaluations like ours provide valuable training material that, when incorporated into algorithm development, enhances platform capabilities for the broader user community. The availability of user-customizable training further positions the technology for continuous improvement as facilities contribute diverse datasets from operational deployments.

We recommend context-dependent implementation strategies: automated sorting for high-volume routine processing where speed and ergonomic benefits are prioritized, combined with manual validation for transgenic line maintenance and accuracy-critical applications. This hybrid workflow leverages automation’s throughput advantages while preserving experimental rigor through targeted human oversight. As commercial platforms mature through iterative refinement informed by field evaluations, the convergence of automation speed with human-level accuracy becomes increasingly achievable, promising to transform zebrafish facility operations while maintaining the scientific standards essential for reproducible research.

## Data Availability

The data presented in this study are contained within the article.

## Supplementary Materials

The following supporting information can be downloaded at: https://www.mdpi.com/article/10.3390/biology15151237/s1, Figure S1: Time-course survival analysis of zebrafish embryos based on automated classification. Video S1: EggSorter Automated Counting supplementary Figure S1.

## Abbreviations

The following abbreviations are used in this manuscript:

AI	Artificial Intelligence
DCNN	Deep Convolutional Neural Networks
dpf	Days Post-Fertilization
EGFP	Enhanced Green Fluorescent Protein
GFP	Green Fluorescent Protein
hpf	Hours Post-Fertilization
IACUC	Institutional Animal Care and Use Committee
IBC	Institutional Biosafety Committee
ML	Machine Learning
MSD	Musculoskeletal Disorder
OECD	Organisation for Economic Co-operation and Development
SD	Standard Deviation
SEM	Standard Error of the Mean
SVM	Support Vector Machine
VAST	Vertebrate Automated Screening Technology
